# Clinical, Biochemical, and Coronary Angiographic Profiling of Symptomatic Premature Coronary Artery Disease in a Gujarati Population: A Cross-Sectional Study

**DOI:** 10.7759/cureus.104213

**Published:** 2026-02-25

**Authors:** Kushal Pujara, Krushan N Yajnik, Bhalendu Vaishnav, Vishal V Bhende

**Affiliations:** 1 Cardiology, Bhanubhai and Madhuben Patel Cardiac Centre, Shree Krishna Hospital and Pramukhswami Medical College, Bhaikaka University, Anand, IND; 2 Cardiology, Sahyadri Super Speciality Hospital, Deccan Gymkhana, Pune, IND; 3 Internal Medicine, Shree Krishna Hospital and Pramukhswami Medical College, Bhaikaka University, Anand, IND; 4 Pediatric Cardiac Surgery, Sri Sathya Sai Sanjeevani Centre for Child Heart Care and Training in Pediatric Cardiac Skills, Navi Mumbai, IND; 5 Pediatric Cardiac Surgery, Bhanubhai and Madhuben Patel Cardiac Centre, Shree Krishna Hospital and Pramukhswami Medical College, Bhaikaka University, Anand, IND

**Keywords:** apolipoproteins, biochemical profile, clinical profile, lipoprotein a, non-conventional risk factors, premature coronary artery disease, southeast asian population, stemi

## Abstract

Background

With the prevalence of coronary artery diseases (CAD) on the rise, especially in the younger population, characterization of non-conventional risk factors remains essential, especially in the inherently predisposed Southeast Asian population. This study aimed at clinical and biochemical profiling in angiographically proven CAD in young Gujarati Indians without conventional risk factors such as tobacco/alcohol consumption.

Methodology

This single-center, descriptive, cross-sectional case series included consecutive Gujarati patients aged ≤45 years presenting with symptomatic, angiographically significant CAD over a 15-month period. Patients with tobacco or alcohol exposure and those with concomitant pre-existing diabetes mellitus and hypertension were excluded. Clinical characteristics, biochemical parameters (glycated hemoglobin, lipid profile, lipoprotein A (LpA), homocysteine, apolipoproteins), and coronary angiographic findings were recorded. Analyses were primarily descriptive, with limited exploratory comparisons.

Results

Overall, 2/4 obese patients (50%) and 3/4 obese patients (75%) were newly diagnosed with dysglycemia and dyslipidemia, respectively. Among patients with single-vessel disease (SVD; n = 16), eight (50%) patients presented with ST-segment elevation myocardial infarction, whereas among those with multi-vessel disease (MVD; n = 6), three (50%) patients presented with non-ST-segment elevation myocardial infarction. Elevated low-density lipoprotein cholesterol levels were observed in 8/16 (50%) patients with SVD and 3/6 (50%) patients with MVD. More than 5/6 (83.3%) patients with elevated LpA had SVD.

Conclusions

The study showed that non-conventional risk factors, such as obesity and family history of CAD, when combined with LpA and lipid profiles, can help in earlier identification of a predisposed individual in a high-risk population.

## Introduction

Globally, the burden of non-communicable diseases (NCDs) is rising significantly, affecting not just the health sector but also the socioeconomic sectors. NCDs were responsible for nearly 74% of the global deaths in 2017, with an increasing trend in the following years [1-3]. As elaborated by the Global Burden of Diseases Collaborative Network [4], there is a significant contribution of NCDs to morbidity and mortality, especially cardiovascular diseases (CVDs), throughout the world. Over the last 30 years, the prevalence of CVDs has increased by more than 50%. The incidence of ischemic heart disease (IHD) has increased by more than 125%. This is associated with similar trends in India, where CVDs and IHD have increased by almost 160% and 200%, respectively.

Although the age bar for labeling premature to a presentation of coronary artery disease (CAD) remains debatable, CVDs account for nearly 60% of the 37.6 million premature deaths in India caused by the four major NCDs [5]. An age limit of ≤45 years for males and ≤55 years for females is routinely used to characterize premature CAD (PCAD) [6,7]. Similarly, age cut-offs of ≤50 years in the Iraqi population [8] and ≤65 years in the Japanese population [9] have been described for genetic studies. A hospital registry-based study from India for evaluation of PCAD defined premature as <55 years for males and <60 years for females, and very premature as <40 years for males and <45 years for females [10]. Of concern, trends similar to those mentioned afore persist even when filtering is done for this younger age group, i.e., 20-54 years of age [4]. Globally, the prevalence of CVDs and IHD in the younger age group has increased by 86% and 105%, respectively, with an associated rise in mortality at 33% and 43%, respectively. At the national level, this data runs in parallel, with a doubling of IHD-related mortality over the last three decades in this subgroup [4].

While it is known that the Southeast Asian population (SEAP) remains at a higher risk of CAD [11], earlier onset [11], more extensive angiographic involvement of the coronaries [11-13], and earlier development of heart failure [11,12], studies describing clinical and angiographic profiling of PCAD remain limited in the Indian setup. SEAP, the largest ethnic group in the world (comprising nearly 2 billion individuals worldwide, including nearly 4 million in the United States [14]), has a higher predisposition to CAD as well as PCAD [14-18]. This holds even in the Indian population, who have an atherosclerotic cardiovascular disease (ASCVD) epidemiology differing significantly from the Western population [18] and develops ASCVD nearly a decade earlier than the latter [19], with the rising burden being a disproportionate one [16]. Furthermore, associations of non-conventional risk factors such as elevated lipoprotein A (LpA) [20] are yet to be established in this high-risk population. Hence, this study aimed at describing the clinical, biochemical, and coronary angiographic profiles of a high-risk ethno-linguistic Gujarati population presenting with symptomatic PCAD in the absence of conventional risk factors such as smoking and coexisting high-risk comorbidities.

## Materials and methods

Study design and setting

This single-center, cross-sectional, observational, and descriptive study was conducted at the Bhanubhai and Madhuben Patel Cardiac Centre, Shree Krishna Hospital, Pramukhswami Medical College, Bhaikaka University, Gujarat, India. The study was conducted over a period of 15 months and included consecutive patients presenting during the study period.

Study population and eligibility criteria

The study population comprised young adult patients of Gujarati ethnicity who presented with symptomatic CAD and underwent coronary angiography during the study period. Patients were eligible for inclusion if they were aged 45 years or younger at the time of the index cardiac event, belonged to the Gujarati ethnic group, were symptomatic at presentation, and had electrocardiographic (ECG), echocardiographic, or clinical indications warranting coronary angiography. Only patients with angiographically significant CAD were included, defined as luminal stenosis ≥70% in any major epicardial coronary artery or ≥50% stenosis of the left main coronary artery.

Patients were excluded if they were older than 45 years, had a history of tobacco consumption in any form (smoked or smokeless), reported alcohol use, had pre-existing concomitant diabetes mellitus and hypertension before presentation, had chronic kidney disease, or declined to provide informed consent. Patients with isolated diabetes mellitus or isolated hypertension without coexistence were not excluded, provided they did not meet other exclusion criteria. A total of 22 patients fulfilled the eligibility criteria and had angiographically significant CAD.

Ethical considerations

The study was conducted in accordance with the ethical principles outlined in the Declaration of Helsinki (1975, revised 2000). Ethical approval was obtained from the Institutional Ethics Committee-2 (IEC-2) of Bhaikaka University (approval ID: IEC/BU/140/Faculty/15/402/2025) dated November 18, 2025. Written informed consent was obtained from all participants before inclusion in the study.

Data collection and clinical evaluation

After screening and enrolment, demographic and epidemiological details were recorded for all patients. A detailed clinical evaluation was performed, including documentation of presenting symptoms, cardiovascular risk factors, personal medical history, past history of IHD, and family history of CAD. Physical examination findings were recorded systematically.

All patients underwent a standard 12-lead ECG at presentation. Transthoracic echocardiography was performed before coronary angiography to assess left ventricular systolic function, regional wall motion abnormalities, and valvular structure and function. Left ventricular ejection fraction (LVEF) was quantified using standard echocardiographic methods.

Biochemical analysis

Venous blood samples were collected from all patients under standardized conditions for biochemical evaluation. Glycemic status was assessed using glycated hemoglobin (HbA1c). Additional biochemical parameters included total homocysteine, complete lipid profile (total cholesterol, low-density lipoprotein (LDL) cholesterol, high-density lipoprotein (HDL) cholesterol, very-low-density lipoprotein (VLDL) cholesterol, and triglycerides), LpA, apolipoprotein A1 (ApoA1), and apolipoprotein B (ApoB). All assays were performed in the institutional laboratory using standardized methods, and results were interpreted using laboratory-specific reference ranges.

The lipid tetrad index was calculated as the product of total cholesterol, triglycerides, and LpA divided by LDL cholesterol, with all values expressed in mg/dL. Blood samples were obtained during the index admission before or at the time of angiographic evaluation.

Coronary angiography and angiographic assessment

All enrolled patients underwent coronary angiography for their respective clinical indications. Angiographic images were reviewed visually to assess the location, severity, and extent of CAD. Lesions causing ≥70% luminal stenosis in major epicardial vessels or ≥50% stenosis in the left main coronary artery were considered significant. Single-vessel disease (SVD) was defined as significant involvement of one major epicardial coronary artery, while multivessel disease (MVD) was defined as involvement of two or more major epicardial vessels. Involvement of sizeable branch vessels in addition to major coronary arteries was considered part of multivessel disease. Chronic total occlusion (CTO) was defined as complete vessel occlusion with absent antegrade flow.

Interventional management and in-hospital treatment

**Figure 1:**
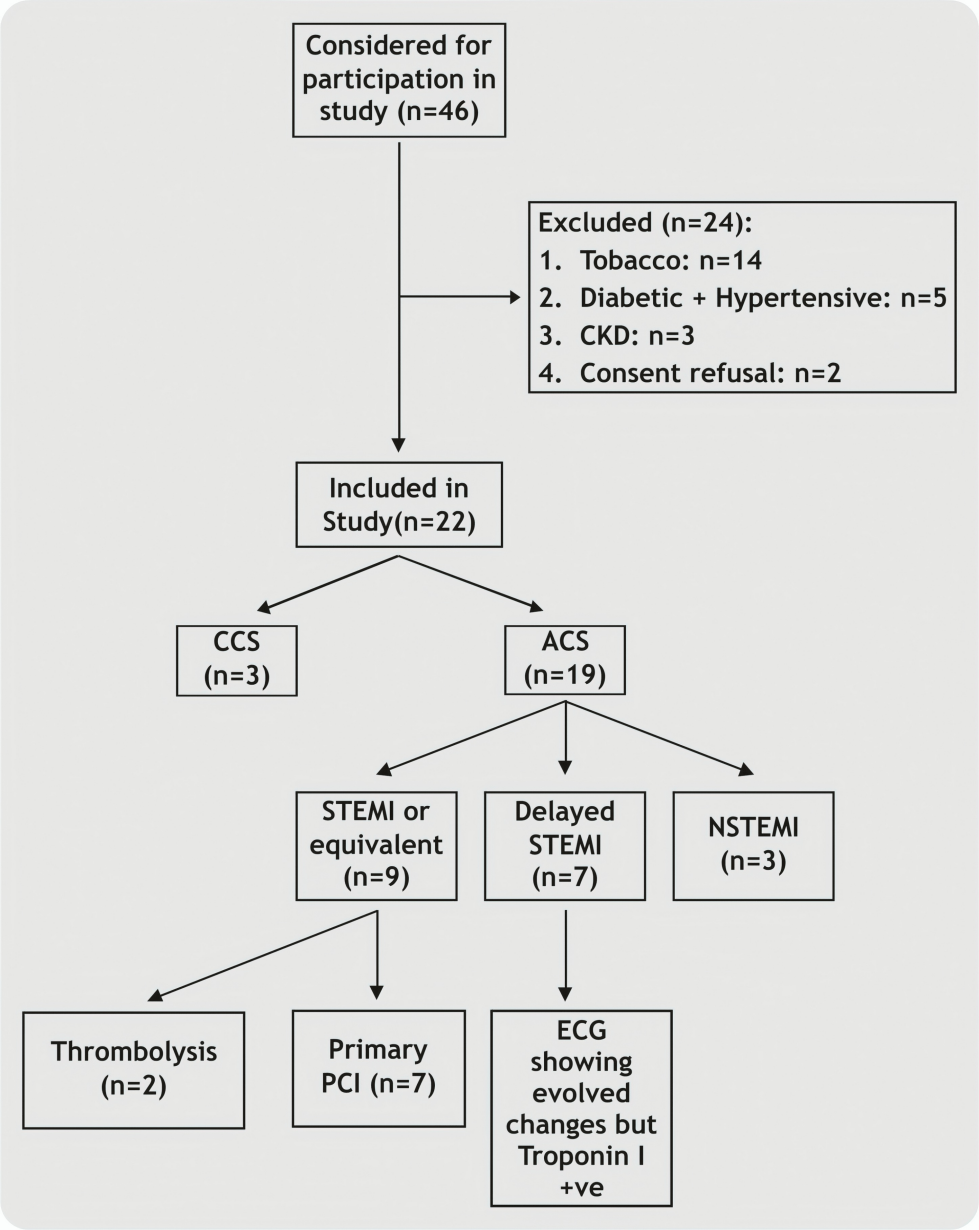
Study methodology. CKD = chronic kidney disease; CCS = chronic coronary syndrome; ACS = acute coronary syndrome; STEMI = ST-elevation myocardial infarction; NSTEMI = non-ST-elevation myocardial infarction; PCI = percutaneous coronary intervention; ECG = electrocardiogram

Percutaneous coronary intervention (PCI) was performed when clinically indicated using contemporary interventional techniques. Details regarding the type of revascularization, including the use of drug-eluting stents, drug-coated balloons, thrombosuction devices, and glycoprotein IIb/IIIa inhibitors, were recorded. Patients who did not undergo percutaneous intervention were managed either medically or referred for coronary artery bypass grafting (CABG) based on angiographic findings and clinical judgment (Figure 1).

Operational definitions

In this study, non-conventional risk factors referred to risk determinants not routinely captured in conventional CAD risk stratification, and operationally included (1) adiposity (body mass index (BMI) categorized using Asian cut-offs); (2) family history of CAD/IHD in first-degree relatives; (3) biochemical markers including LpA, total homocysteine, and apolipoproteins (ApoA1, ApoB), in addition to conventional lipid fractions and HbA1c; and (4) delayed presentation ST-elevation myocardial infarction (STEMI) was defined as evolved ST-elevation changes with positive hs-troponin I beyond the primary reperfusion window.

Statistical analysis

Data were analyzed using both descriptive and inferential statistical methods. Continuous variables were assessed for normality using the Shapiro-Wilk test and are presented as mean ± standard deviation (SD) or median with interquartile range (IQR), as appropriate. Categorical variables are expressed as numbers and percentages. Given the small sample size and non-normal distribution of several variables, non-parametric statistical tests were employed. Comparisons between categorical variables were performed using Fisher’s exact test, while continuous variables between two groups were compared using the Mann-Whitney U test. All statistical tests were two-tailed, and a p-value <0.05 was considered statistically significant. No multivariable adjustment was performed due to the limited sample size. Inferential analyses were interpreted cautiously, given the exploratory nature of the study.

## Results

Clinical profiling

*Epidemiological* *Profile*

**Figure 2:**
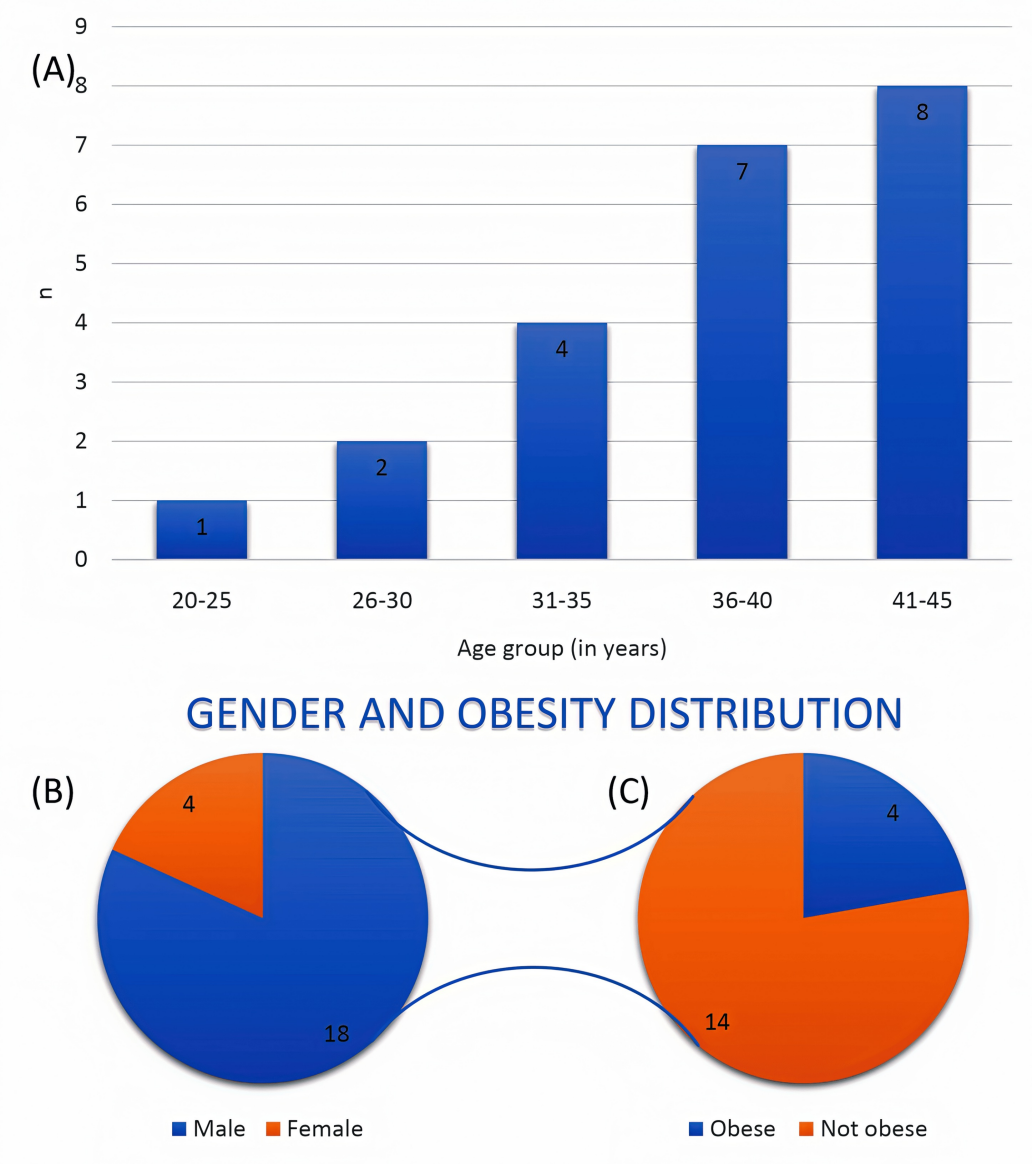
Epidemiological profile. (A) Age group-wise distribution of patients, expressed as the number of patients (n) across predefined age categories (total cohort, n = 22). (B) Gender distribution of the study population showing 18 (81.8%) males and 4 (18.2%) females (total cohort, n = 22). (C) Obesity distribution among male patients only (n = 18), showing 4 (22.2%) obese males and 14 (77.8%) non-obese males. No female participants met the body mass index criterion for obesity; therefore, females were not included in Panel C.

The patients ranged from ages 23 to 45 years. There was a male preponderance, with 18 of 22 (81.8%) patients being male. All obese (BMI = >23 kg/m²) patients (n = 4) were males (Figure 2).

Presenting Complaints, Clinical Examination, and Diagnosis

All patients were symptomatic on presentation at the index cardiac event, with angina being the predominant presentation. Three patients with both angina and dyspnea on exertion were diagnosed with chronic coronary syndrome (CCS), two of whom had a positive treadmill test (TMT), and the third had an ECG showing changes of an evolved anterior wall infarction, with echocardiographic findings corroborating the ECG findings.

Furthermore, 19 (86%) patients had presented with an acute coronary syndrome (ACS), of whom nine had STEMI or a STEMI equivalent. Of these nine STEMI/STEMI equivalents, three patients had an acute anterior wall infarction, one had a new-onset left bundle branch block, and five patients had inferior and/or posterior wall STEMI. Two patients with posterior wall infarctions underwent pharmacoinvasive therapy with thrombolysis followed by coronary angiography and PCI.

Additionally, of the 19 patients with ACS, seven presenting with angina had evolved changes in their ECGs. However, their high-sensitivity troponin I test (hsTropI) was positive, and they were classified as delayed presentation STEMI. Hence, they were advised to undergo coronary angiography. Similarly, three out of the 19 ACS patients presented with non-specific ST-T changes on their ECGs, but a positive hsTropI test. They were diagnosed as non-STEMI (NSTEMI) and were advised to undergo coronary angiography. None of the patients had any positive remarkable physical examination findings localizing to the cardiovascular system.

Comorbid Conditions

Patients with coexistent diabetes mellitus and hypertension were excluded (n = 5). None of the patients was hypertensive before the index cardiac event. Two patients were newly diagnosed with hypertension (one of whom was also newly diagnosed with pre-diabetes) and were discharged on antihypertensive medications. With regards to diabetes mellitus, three patients were known diabetics before their presentations, none of whom were on insulin therapy. Only one of them had a fairly controlled HbA1c of 6.4; the other two had hyperglycemia without ketosis on presentation. A staggering 59% of the patients (n = 13) were found to have elevated HbA1c levels, with two patients being diagnosed with diabetes mellitus, and the remaining 11 patients categorized as prediabetics. The two newly diagnosed diabetics were discharged on medical management for the same.

Two patients had presented with new symptoms, but had a previous history of IHD. The first of these patients (Patient A henceforth), now a 60-year-old male, but a 39-year-old with newly diagnosed diabetes mellitus at the time of the index cardiac event, presented with TMT-positive CCS with old evolved inferior wall ST-T changes on ECG. He had a history of plain old balloon angioplasty to the left circumflex (LCx) artery during the index event, followed by CABG surgery at the age of 54 years. Hence, he was advised to undergo a repeat coronary angiography. The second of these two patients, now a 39-year-old non-diabetic, non-hypertensive male, had undergone a percutaneous transluminal coronary angioplasty to the left anterior descending (LAD) artery at the age of 27 years. He had also presented with a TMT-positive CCS with a normal resting ECG without any gross echocardiographic changes, and was advised to undergo coronary angiography for the same. Two patients had prior revascularization procedures and were included in the descriptive analysis.

Family History

Six patients had a significant positive family history of IHD. As a common factor, all six patients had fathers with a history of CAD. Patient A (as mentioned above) had a very strong family history, with the father having PCAD at the age of 39 years, elder brother at the age of 35 years, and two paternal uncles at ages 41 years and 54 years. Another patient, who was now newly diagnosed with prediabetes and had presented with STEMI involving the inferior wall, had a positive history of a father suffering from an acute cardiac event and subsequently succumbing to it at the age of 52 years. Yet another patient, now presenting with hsTropI-positive ACS, had a positive family history of CAD in the father and paternal uncle. None of the patients had any positive cardiac history on their maternal sides.

Electrocardiography and Echocardiography

Infarction and ischemia-related changes have been described above. In addition, two patients had runs of malignant ventricular arrhythmias. The first of these two patients had runs of ventricular tachycardia (VT) followed by ventricular fibrillation (VF) and cardiac arrest. The patient was revived after a short cardiopulmonary resuscitation cycle, including DC cardioversion, and subsequently underwent primary PCI. The second patient had multiple runs of ill-sustained VT, managed medically.

**Figure 3:**
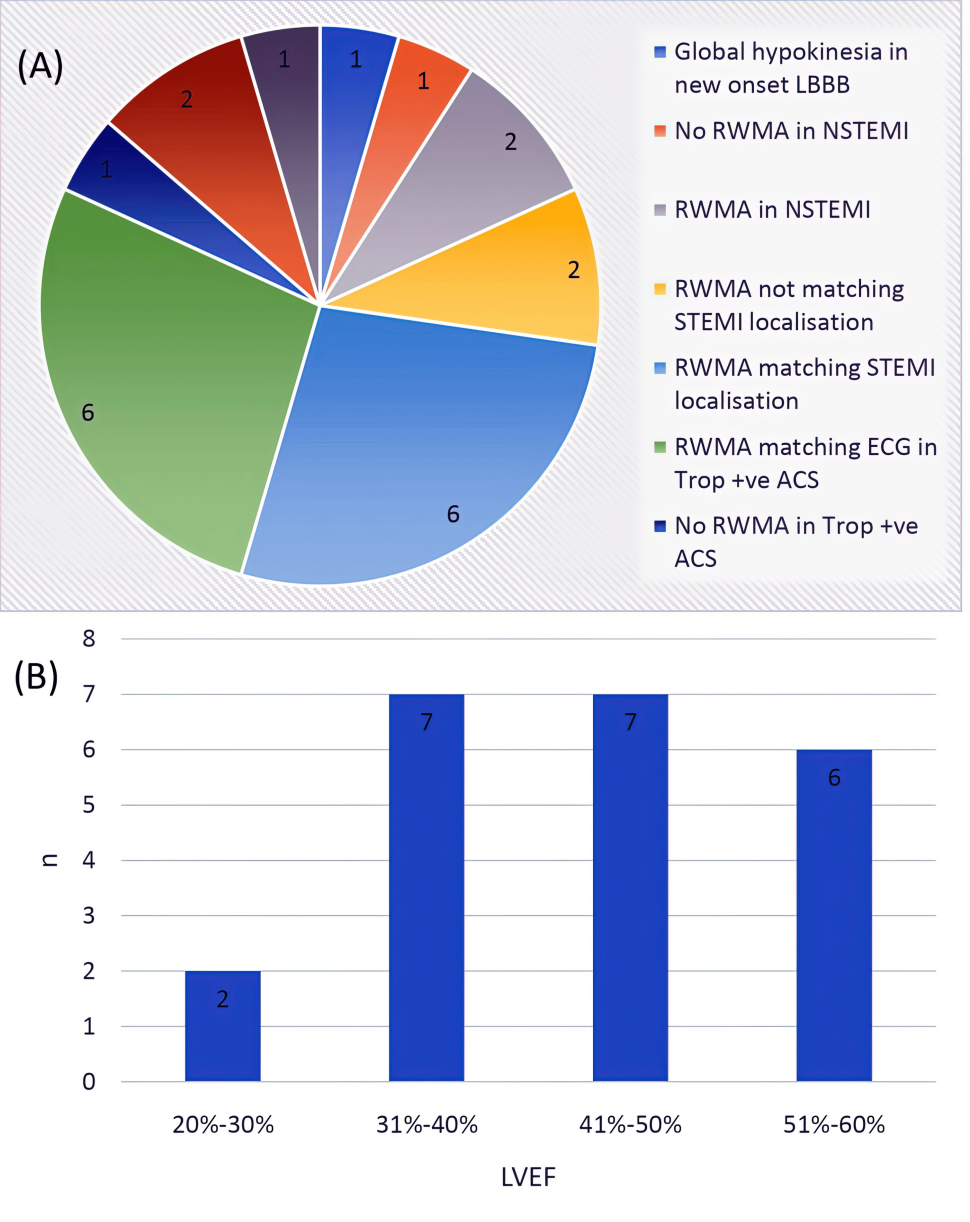
Echocardiographic profile. (A) Various regional wall motion changes among the patients. (B) Distribution of patients within various LVEF ranges. ACS = acute coronary syndrome; LBBB = left bundle branch block; LVEF = left ventricular ejection fraction; NSTEMI = non-ST-segment elevation myocardial infarction; RWMA = regional wall motion abnormality; STEMI = ST-segment-elevation myocardial infarction; Trop = troponin I; ECG = electrocardiogram

Regarding echocardiography (Figure 3), 11 (50%) patients had LVEF ≤45%. Five patients had no regional wall motion abnormality, of whom two had STEMI, and one had evolved anterior wall myocardial infarction-related changes on ECG. Seven out of the nine patients with STEMI/STEMI equivalents had regional wall motion abnormality corresponding to the respective ECG territories. Seven patients were noted in the range 31% to 40% and 41% to 50%, each. None of the patients had any significant valvular lesions, although six patients were found to have a clinically non-significant mild mitral regurgitation.

Biochemical profiling

Patients underwent blood analysis for biochemical parameters, which have been hypothesized to be associated with cardiac morbidity and mortality. Their deviations from expected results are presented in Table 1.

**Table 1:** Biochemical parameters and their deviations.

Biochemical parameter	Category	Reference range	Frequency, N (%)
Glycosylated hemoglobin	Non-diabetic	<5.7%	6 (27.3%)
Glycosylated hemoglobin	Prediabetic	5.8–6.3%	11 (50.0%)
Glycosylated hemoglobin	Diabetic	≥6.4%	5 (22.7%)
Homocysteine	Within normal limits	5–15 µmol/L	19 (86.4%)
Homocysteine	Elevated	>50 µmol/L	3 (13.6%)
Total cholesterol	Within normal limits	<200 mg/dL	16 (72.7%)
Total cholesterol	Elevated	≥200 mg/dL	6 (27.3%)
Low-density lipoprotein cholesterol	Within normal limits	<130 mg/dL	11 (50.0%)
Low-density lipoprotein cholesterol	Elevated	≥130 mg/dL	11 (50.0%)
Very-low-density lipoprotein cholesterol	Within normal limits	<35 mg/dL	17 (77.3%)
Very-low-density lipoprotein cholesterol	Elevated	≥35 mg/dL	5 (22.7%)
High-density lipoprotein cholesterol	Within normal limits	40–60 mg/dL	13 (59.1%)
High-density lipoprotein cholesterol	Reduced	<40 mg/dL	9 (40.9%)
Triglycerides	Within normal limits	<150 mg/dL	14 (63.6%)
Triglycerides	Elevated	≥150 mg/dL	8 (36.4%)
Lipoprotein A	Within normal limits	<0.3 g/L	13 (68.4%)
Lipoprotein A	Elevated	≥0.3 g/L	6 (31.6%)
Apolipoprotein A1	Within normal limits	0.7–1.3 g/L	12 (66.7%)
Apolipoprotein A1	Elevated	>1.3 g/L	6 (33.3%)
Apolipoprotein B	Within normal limits	<1.3 g/L	19 (100%)
Apolipoprotein B	Elevated	≥1.3 g/L	0 (0%)

Glycated Hemoglobin

A total of five patients had diabetic range levels (HbA1c ≥6.4%), of whom two were newly diagnosed as diabetics. Furthermore, 11 patients had prediabetic range levels (HbA1c 5.7% to 6.3%), all of whom were newly diagnosed prediabetics. Newly elevated HbA1c refers to newly detected prediabetes or diabetes during the index admission.

Homocysteine

Hyperhomocysteinemia was defined as >15 µmol/L; levels >50 µmol/L were categorized as markedly elevated. Three patients were found to have significant hyperhomocysteinemia (>50 µmol/L). However, if the standard normative range (5-15 µmol/L) was considered, an additional eight patients could be considered as having hyperhomocysteinemia.

Lipid Profile

**Figure 4:**
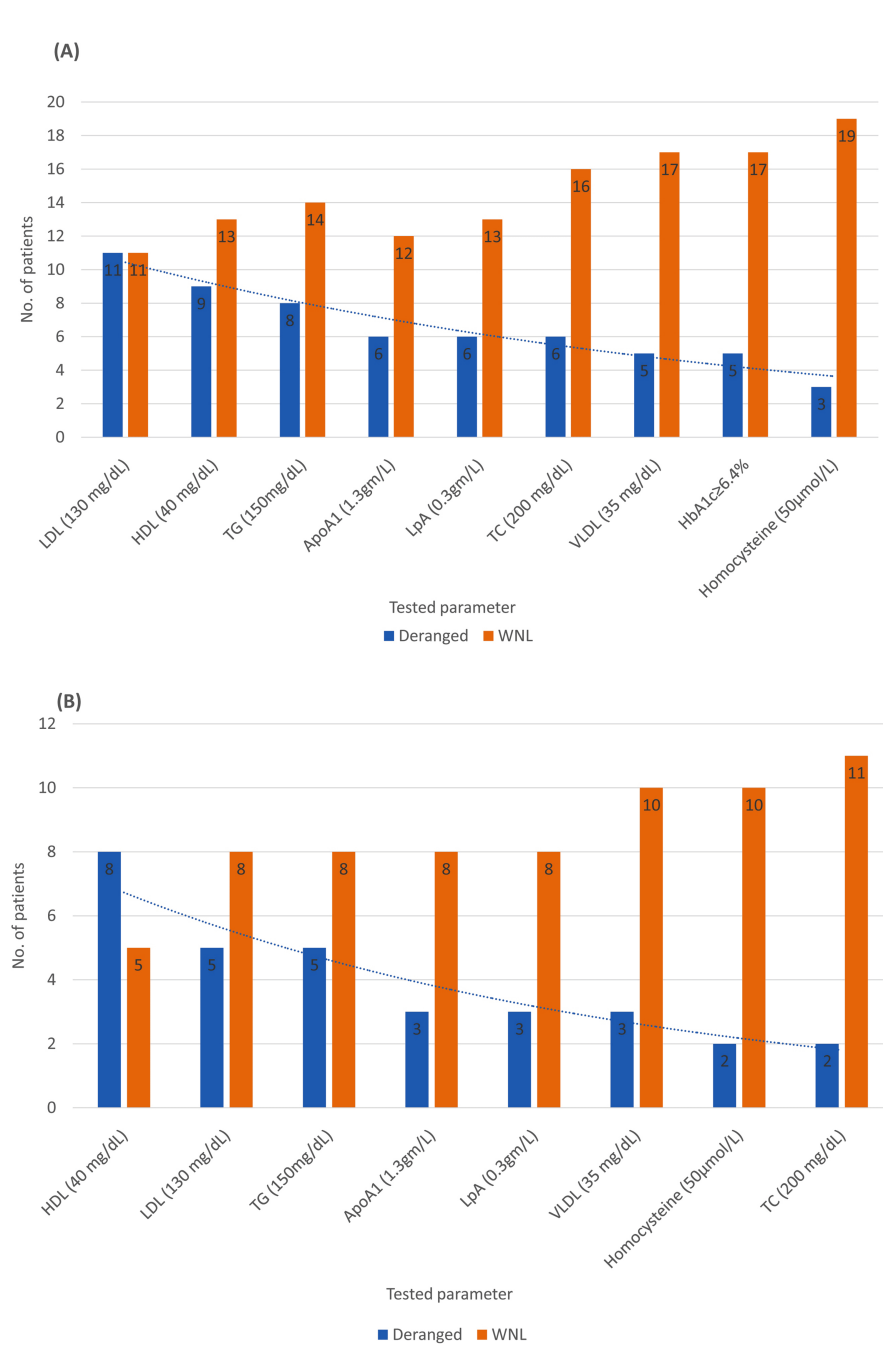
(A) Biochemical profile in 22 patients. (B) Biochemical profile of 13 patients with newly deranged HbA1c levels. Values in parentheses denote the cut-off reference ranges. ApoA1 = apolipoprotein A1; HbA1c = glycated hemoglobin; HDL = high-density lipoprotein; LDL = low-density lipoprotein; LpA = lipoprotein A; MVD = multivessel disease; SVD = single-vessel disease; TC = total cholesterol; TG = triglycerides; VLDL = very-low-density lipoprotein; WNL = within normal limits

Overall, 17/22 (77.3%) patients had at least one lipid abnormality, the most common deviation being elevated LDL (50%), followed by reduced HDL (41%) and elevated triglycerides (36%). Surprisingly, only five patients had a completely normal lipid profile, two of whom were previously known diabetics on medical management, and two were newly diagnosed prediabetics (Figures 4A, 4B).

Of the two newly diagnosed diabetics, one had reduced HDL levels, and the other had hypertriglyceridemia and high LDL levels. Furthermore, of the 11 patients with newly diagnosed prediabetic range HbA1c levels, two had completely normal lipid profiles, two had elevated total cholesterol, three had elevated LDL, seven had low HDL, two had elevated VLDL, and four had elevated triglycerides.

The Lipid Tetrad Index (LTI) (calculated as total cholesterol × triglycerides × LpA/HDL; all values in mg/dL) is a comprehensive marker for dyslipidemia. The mean LTI in the current study was 20,464.6, with six (27.3%) patients having an LTI higher than 20,000.

Apolipoproteins A1 and B

As all patients had ApoB within the normal range, the focus will be on ApoA1. Six patients had elevated ApoA1 levels (>1.3 g/L). This included two out of the three previously known diabetic patients, two newly diagnosed prediabetics, and one newly diagnosed diabetic patient. One had a normal HbA1c level. Among 11 newly diagnosed prediabetics, two had elevated ApoA1 levels. The mean ApoB/ApoA1 ratio was found to be 0.6 (n = 18).

Lipoprotein A

A total of six patients had elevated LpA, including two patients with newly diagnosed prediabetic level HbA1c, as well as one previously known and one newly diagnosed diabetic.

Clinico-biochemical associations

**Figure 5:**
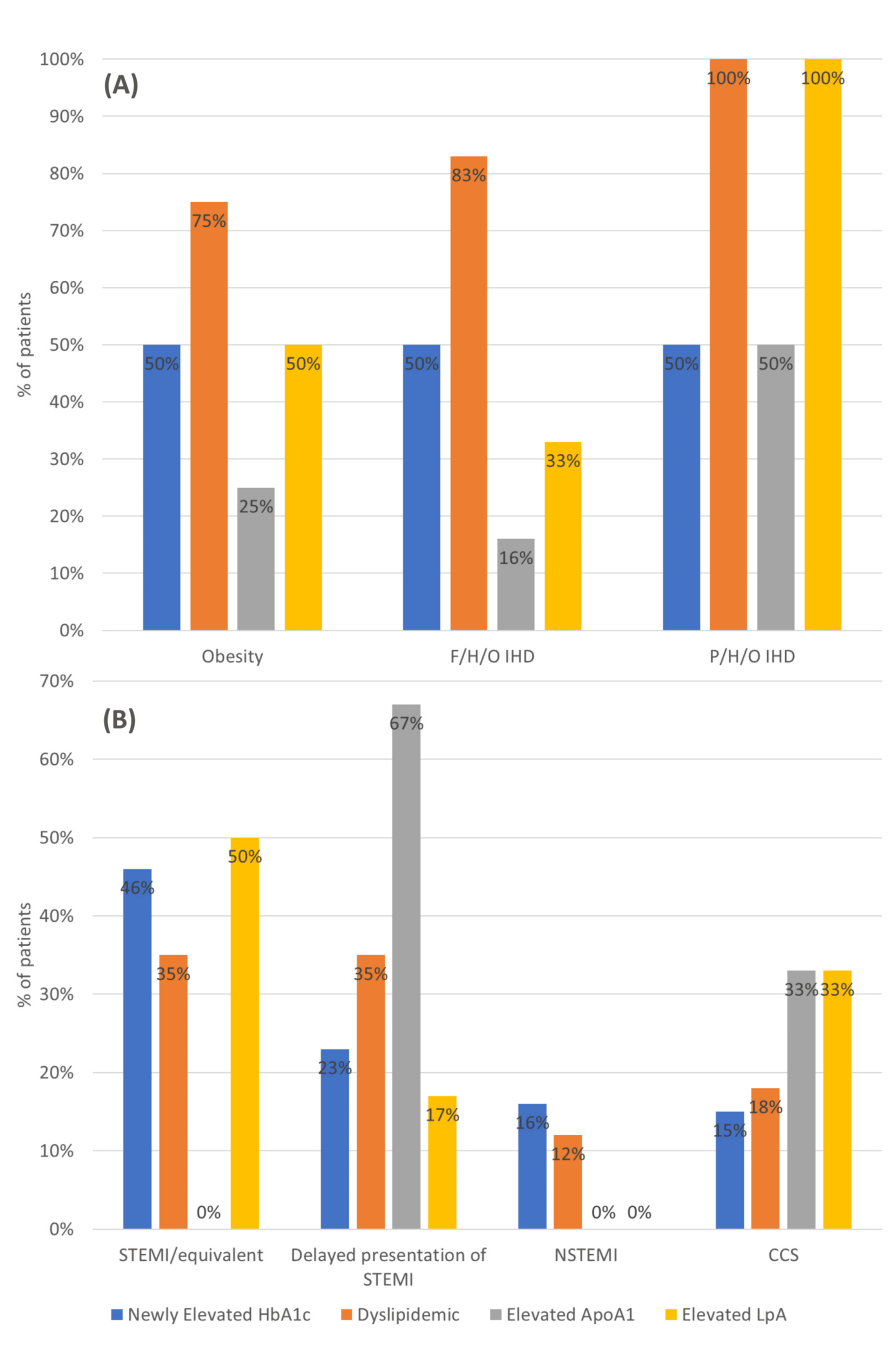
(A) Clinico-biochemical associations. (B) Clinical presentation and biochemical profile. (A) Distribution of biochemical abnormalities among patients with obesity, a family history of ischemic heart disease, and a personal history of ischemic heart disease. (B) Distribution of biochemical abnormalities according to clinical presentation, including ST-segment elevation myocardial infarction or equivalent, delayed presentation of ST-segment elevation myocardial infarction, non-ST-segment elevation myocardial infarction, and chronic coronary syndrome. STEMI = ST-segment elevation myocardial infarction; NSTEMI = non-ST-segment elevation myocardial infarction; CCS = chronic coronary syndrome; IHD = ischemic  heart disease; F/H/O = family history of; P/H/O = past history of; ApoA1 = apolipoprotein A1; HbA1c = glycated hemoglobin; LpA = lipoprotein A

The detailed clinico-biochemical associations are elaborated in Table 2 and Figure 5. Some significant findings are discussed below.

**Table 2:** Clinico-biochemical and clinico-angiographic associations. Subgroups are not mutually exclusive; totals may exceed 22. ACS = acute coronary syndrome; ApoA1 = apolipoprotein A1; CCS = chronic coronary syndrome; F/H/O = family history of; IHD = ischemic heart disease; NSTEMI = non-ST-segment elevation myocardial infarction; P/H/O = past history of; STEMI = ST-segment elevation myocardial infarction; WMA = wall motion anomaly

Serial number	Patient subset	Obesity		F/H/O IHD		P/H/O IHD		Diagnosis on presentation				Regional/Global WMA		Ejection fraction			
Clinico-biochemical associations																	
		Yes (n = 4)	No	Yes (n = 6)	No	Yes (n = 2)	No	STEMI / equivalent (n = 9)	Delayed presentation of STEMI (n = 7)	NSTEMI (n = 3)	CCS (n = 3)	Yes (n = 17)	No	20%–30%	31%–40%	41%–50%	51%–60%
1	Newly diagnosed prediabetic (n = 11)	2	9	1	10	0	11	5	3	2	1	8	3	2	2	4	3
2	Newly diagnosed diabetic (n = 2)	0	2	2	0	1	1	1	0	0	1	2	0	0	0	2	0
3	Dyslipidemia (of any parameter) (n = 17)	3	14	5	12	2	15	6	6	2	3	12	5	2	2	7	6
4	Elevated ApoA1 (n = 6)	1	5	1	5	1	5	0	4	0	2	5	1	1	2	1	2
5	Elevated lipoprotein A (n = 6)	2	4	2	4	2	4	3	1	0	2	3	3	0	1	2	3
Clinico-angiographic associations																	
1	Single-vessel disease (n = 16)	3	13	5	11	2	0	8	6	0	2	12	4	1	5	5	5
2	Multivessel disease (n = 6)	1	5	1	5	0	0	1	1	3	1	5	1	1	2	2	1

Elevated Glycated Hemoglobin

Only 2 of the 13 patients with newly elevated HbA1c levels were found to be obese. Conversely, 50% of the obese patients were newly diagnosed as prediabetics. Interestingly, 50% (n = 3) of the patients with a family history of IHD were found to have elevated HbA1c. Overall, 45% of patients with newly elevated HbA1c levels presented with STEMI/STEMI-equivalent ECG findings.

Dyslipidemia

Of the 17 patients with dyslipidemia, 17% (n = 3) were obese, and nearly 30% (n = 5) had a family history of IHD. Conversely, 75% of obese patients and >83% of patients with a family history of IHD were also found to be dyslipidemic. Both patients with a previous history of ischemic cardiac events were found to be dyslipidemic. While numerically both STEMI and delayed presentation of STEMI remained equal, the latter group constituted a larger proportion, with 85% of the total delayed presentation group.

Elevated Apolipoprotein A1

Of the six patients with elevated ApoA1, one patient was found to be obese and with a family history of IHD, each, thereby constituting 25% and 16% of their respective groups. However, none of them presented with STEMI/STEMI equivalents or NSTEMI, with delayed presentation of STEMI being the most common presentation (66%) in this group. Patient A, with a family history of IHD, was found to have elevated ApoA1.

Elevated Lipoprotein A

Overall, 33% of the patients with elevated LpA were obese, which constituted 50% of the obese patients. Interestingly, two patients with a personal and family history of IHD each were found to have elevated LpA. Hence, 100% of the patients with personal history (including Patient A) and 33% of patients with a family history of IHD had elevated LpA. STEMI/STEMI-equivalent ECG remained the most common clinical presentation (50%). Conversely, 33% (n = 3) of patients with STEMI/STEMI equivalents had elevated LpA.

Angiographic profiling

All 22 patients underwent coronary angiography for their respective indications. Overall, 16 patients had SVD (including one patient each with recanalized vessel and non-obstructive CAD) and six patients had MVD. Left anterior descending (LAD) was the most commonly involved artery (n = 13; 59%), followed by the right coronary artery (RCA) (n = 5; 23%) and LCx (n = 4; 18%). Among branch vessels, the diagonal artery was involved in two patients, the obtuse marginal artery in three patients, and the posterior descending artery in one patient (where it was a major branch of a super-dominant RCA).

The LAD was encountered as a part of MVD in five patients, of whom two patients had a coexistent chronic total occlusion of the RCA, two had coexistent LCx disease, and one was involved in a Medina 1,1,1 bifurcation with a sizeable diagonal branch.

Lesions not warranting PCI were noted in three patients, all of whom were advised medical management. One of them showed slow flow and recanalized the RCA, which was thrombolyzed for STEMI of the inferoposterior wall. The other showed a non-obstructive CAD of a major posterior descending artery branch of a super-dominant RCA in a patient with delayed presentation of STEMI, with ECG showing evolved inferior wall myocardial Infarction. The third patient, also a delayed presentation with evolved anterior wall infarction, had complete thrombotic occlusion of the proximal LAD.

Clinico-angiographic associations

Single-Vessel Disease

Among the 16 patients with SVD, three were obese, and five had a family history of IHD. Both patients with a previous history of IHD had SVD. With respect to comorbid conditions, one patient was newly diagnosed with hypertension and diabetes mellitus, each. Eight of the 11 newly diagnosed prediabetics had SVD. Overall, 50% of the SVD patients presented with STEMI/STEMI equivalents, thereby constituting nearly 90% of the clinical presentation.

Multivessel Disease

A total of six patients had MVD, of whom one patient was obese and had a family history of IHD each. One patient was newly diagnosed with hypertension and diabetes each, and 3 were newly diagnosed with prediabetes. All patients presenting with NSTEMI had MVD, constituting 50% of the MVD clinical presentations.

Angiographic-biochemical associations

**Figure 6:**
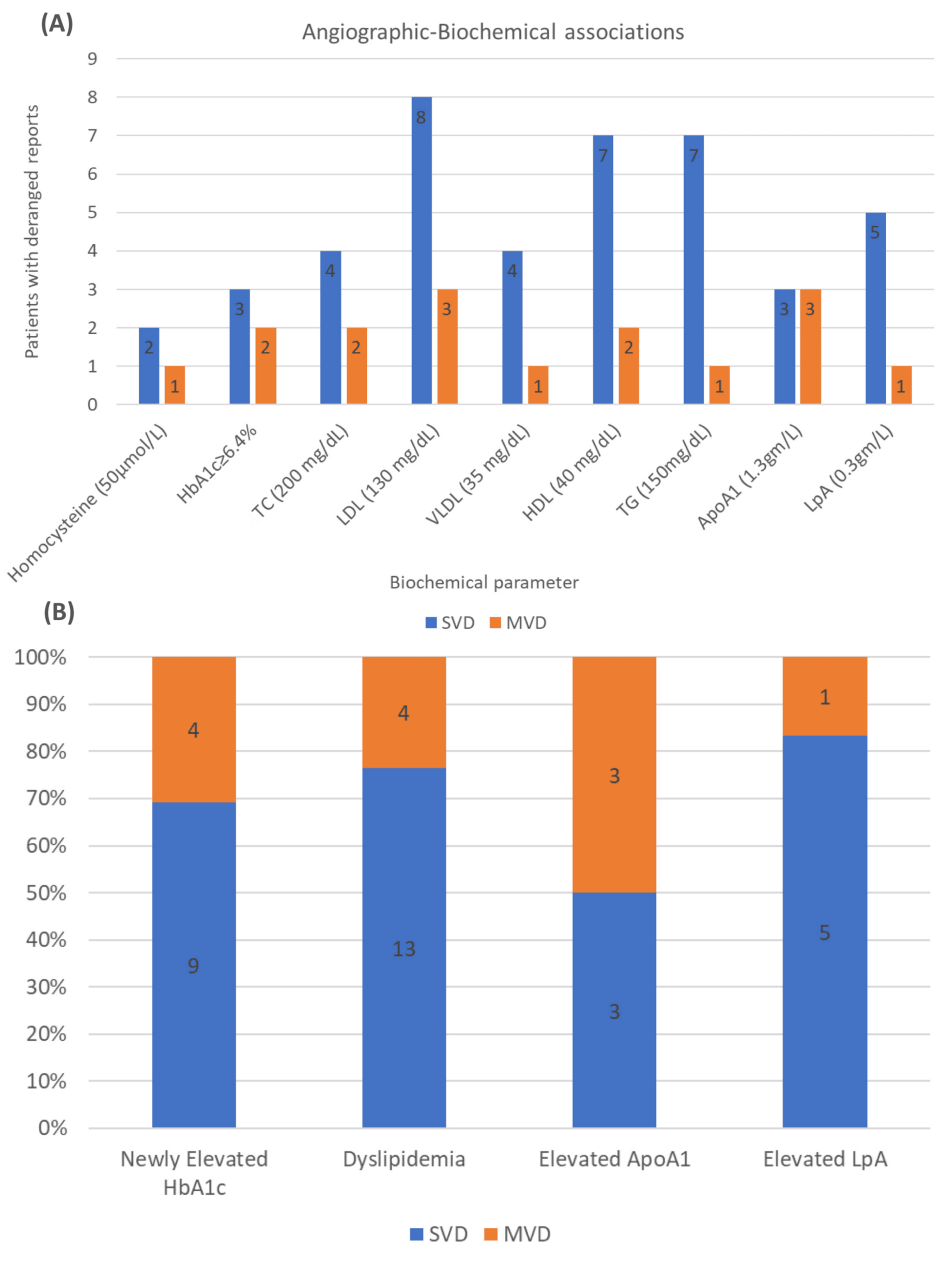
(A) Angiographic-biochemical associations. (B) Biochemical parameters. Values in parentheses denote the cut-off reference ranges. ApoA1 = apolipoprotein A1; HbA1c = glycated hemoglobin; HDL = high-density lipoprotein; LDL = low-density lipoprotein; LpA = lipoprotein A; MVD = multivessel disease; SVD = single-vessel disease; TC = total cholesterol; TG = triglycerides; VLDL = very-low-density lipoprotein

Angiographic-biochemical associations are presented in Table 3 and Figure 6.

**Table 3:** Angiographic-biochemical associations. ApoA1 = apolipoprotein A1; HbA1c = glycated hemoglobin; LpA = lipoprotein A; MVD = multivessel disease; SVD = single-vessel disease

Vessel involvement	Elevated HbA1c (known diabetics + newly diagnosed) (n = 16)	Newly elevated HbA1c (n = 13)	Dyslipidemia (n = 17)	Elevated ApoA1 (n = 6)	Elevated LpA (n = 6)
SVD (n = 16)	11	9	13	3	5
MVD (n = 6)	5	4	4	3	1

Single-Vessel Disease

Elevated LDL was the most common biochemical derangement in patients with SVD (n = 8/16; 50%), followed by elevated triglycerides and low HDL levels, with seven (44%) patients each. Notably, 50% of the patients with elevated ApoA1 and 83% of the patients with elevated LpA had SVD.

Multivessel Disease

Overall, 50% of the patients with MVD had elevated LDL and ApoA1 levels each. In contrast to SVD findings, elevated triglycerides and LpA were noted only in 16% of the patients each.

Outcomes

All 22 patients were discharged with stable hemodynamics. The two newly diagnosed diabetics, as well as hypertensive patients, were discharged with oral medical management with a plan of adjustment on follow-up. Patients with newly diagnosed prediabetics were counseled regarding non-pharmacological means of glycemic control, with a plan to check fasting as well as postprandial sugar levels at the one-month follow-up.

Interventional Management

Regarding revascularization, 17 patients underwent PCI. Among the remaining five patients, two underwent CABG for MVD, and three underwent medical management. Of the 17 patients who underwent PCI, 13 had only drug-eluting stent (DES) implantation, three had drug-coated balloon (DCB) management, and one had main vessel DES + side branch DCB. Seven patients each had a concomitant use of a thrombosuction device and GpIIbIIIa inhibitor (Tirofiban).

Medical Management

All patients were discharged on aspirin and ticagrelor-based dual antiplatelet therapy along with statins. The 11 patients with LVEF ≤45% were also started on guideline-directed medical therapy, including cardio-selective beta-blockers, diuretics, angiotensin-converting enzyme (ACE) inhibitors, and sodium-glucose cotransporter 2 (SGLT2) inhibitors, with a plan for dose titration on follow-up. The two patients, who were planned for CABG, were kept on aspirin-based single antiplatelet therapy with statin therapy along with low-molecular-weight heparin.

Inferential statistical analysis

A statistically significant association was observed between clinical presentation and angiographic severity, with STEMI being more frequent in SVD and NSTEMI being more frequent in MVD (Fisher’s exact test: odds ratio (OR) = Inf; continuity-corrected OR = 39.67, 95% confidence interval (CI) = 1.28-1,229.94; p = 0.018). The infinite OR reflects perfect separation of outcomes across angiographic categories; a continuity-corrected OR with 95% CI has therefore been additionally reported for interpretability.

Obesity was not significantly associated with the presence of dyslipidemia (Fisher’s exact test: OR = 0.86; continuity-corrected OR = 0.72, 95% CI = 0.08-6.44; p = 1.000), nor with dysglycemia or angiographic severity (all p > 0.05). No statistically significant associations were observed between angiographic severity and elevated LDL cholesterol, elevated LpA, or elevated ApoA1. Given the exploratory nature and limited sample size, inferential findings should be interpreted cautiously and are intended to be hypothesis-generating.

## Discussion

The current study profiled clinical, biochemical, and angiographic findings in the high-risk Gujarati subgroup of the SEAP presenting with symptomatic PCAD without any conventional risk factors and attempted to determine the associations among them. Clinically, ACS remained the predominant manifestation, with dyslipidemia, newly elevated HbA1c levels, and elevated LpA and ApoA1 levels being the salient biochemical findings. SVD with LAD involvement was the most common coronary angiography finding. The prevalence, mortality, and disease burden findings were reviewed in the general population (Table 4), as well as in the younger (age 20 years to 54 years) population, and trends were noted (Table 5).

**Table 4:** Highlights of NCD burden internationally and nationally across all ages. Summarized from the Global Burden of Diseases Collaborative Network [4] accessed in September 2025. Units of measurement: Number of cases, deaths, and DALY: in millions; age-standardized prevalence of death rates; and DALY: per 1 lakh population. CVD = cardiovascular diseases; DALY = disability-adjusted life years; IHD = ischemic heart disease; NCD = non-communicable diseases

Variable			NCD			CVD			IHD		
			Year 1990	Year 2021	% Change	Year 1990	Year 2021	% Change	Year 1990	Year 2021	% Change
Global scenario	Prevalence	Number of cases	4,798.9	7,255.1	51%	289.5	612.1	111%	112.2	254.3	127%
		Age-standardized prevalence	91,111	91,034	-0.1%	7,116	7,178	0.9%	2,904	2,946.4	1%
	Mortality	Deaths	26.8	43.8	63%	12.3	19.4	58%	5.4	9	67%
		Age-standardized death rate	734.2	529.7	-28%	358.1	235.18	-34%	158.9	108.7	-32%
	Disease burden	DALY	1,150.2	1,727.2	50%	297.5	428.3	44%	119.2	188.4	58%
		Age-standardized DALY	25,885	20,783	-20%	7,550.2	5,056	-33%	3,107.6	2,212.2	-29%
Southeast Asia	Prevalence	Number of cases	1,171	1,908.1	63%	49.8	127.8	157%	23.42	67.6	189%
		Age-standardized prevalence	92,026	91,767	-0.3%	6,657	6,959.1	4.5%	3,554	3,823.8	8%
	Mortality	Deaths	4.6	9.8	113%	1.9	4.6	142%	0.8	2.2	175%
		Age-standardized death rate	694.7	606	-13%	316.7	280.1	-11.5%	130	136.8	5%
	Disease burden	DALY	244.5	431.4	76%	59.1	116	96%	23.5	56.4	140%
		Age-standardized DALY	26,255.5	22,643.9	-14%	7,629	6,345.2	-17%	3,088.8	3,058.7	-1%
India	Prevalence	Number of cases	767.4	1,311.1	71%	34.1	89.2	161%	17.9	51.9	190%
		Age-standardized prevalence	92,153	92,134	-0.02%	6,949.5	7,339.4	6%	4,110.9	4,458.1	8%
	Mortality	Deaths	2.9	6.5	124%	1.2	2.9	142%	0.6	1.6	166%
		Age-standardized death rate	662.7	603.1	-9%	287.5	267.6	-7%	137.9	151.2	10%
	Disease burden	DALY	158	289.5	83%	36.9	74.5	102%	16.8	41.3	146%
		Age-standardized DALY	25,842	22,776	-12%	7,128.4	6,151.1	-14%	3,321.5	3,400	2%

**Table 5:** Highlights of NCD burden internationally and nationally in the age group of 20 years to 54 years. Summarized from the Global Burden of Diseases Collaborative Network [4] accessed in September 2025. Units of measurement: Number of cases, deaths, and DALY: in millions; age-standardized prevalence of death rates; and DALY: per 1 lakh population. CVD = cardiovascular diseases; DALY = disability-adjusted life years; IHD = ischemic heart disease; NCD = non-communicable diseases

Variable			NCD			CVD			IHD		
			Year 1990	Year 2021	% Change	Year 1990	Year 2021	% Change	Year 1990	Year 2021	% Change
Global scenario	Prevalence	Number of cases	2,311.7	3,656.6	58%	79.2	147.4	86%	19.8	40.7	105.5%
	Mortality	Deaths	4.1	5.4	32%	1.5	2	33%	0.7	1	43%
	Disease burden	DALY	402.7	604.1	50%	75.4	97.6	29%	30.6	44.5	45%
Southeast Asia	Prevalence	Number of cases	543.4	1,014.9	87%	17.1	36.2	112%	4.9	11.8	141%
	Mortality	Deaths	1	1.6	60%	0.4	0.7	75%	0.2	0.4	100%
	Disease burden	DALY	99.3	172.2	73%	22	34.5	57%	9.6	17.9	86%
India	Prevalence	Number of cases	355.6	696.3	96%	11.7	25.5	118%	3.8	9.1	139%
	Mortality	Deaths	0.7	1.1	57%	0.3	0.5	67%	0.15	0.3	100%
	Disease burden	DALY	65.6	116.6	78%	14	22.4	60%	6.9	13.1	90%

Clinical profiling

The current study reported a male preponderance of PCAD (82%), which was comparable to that reported by Muthu et al. (87%) [16], Bana et al. (86.3%) [21], and Prajapati et al (90%) [22], but lower than that reported by Kumar et al. (97.8%) [23]. However, this is in contrast to that reported by Sharma et al. [10], who reported a significantly higher prevalence of PCAD in women (33%) compared to men (29.2%). Obesity was reported at 18% in the current study, which is fairly higher than that reported by the aforementioned authors at 7.2% [16] and 5.5% [22]. A family history of IHD was reported in the current study (27.3%), which is lower than Bana et al. (41.9%) [21] but higher than that reported by Kumar et al. (19%) [23], Prajapati et al. (17%) [22], and Muthu et al. (11%) [16]. Smoking and/or tobacco consumption remained a risk factor that was most frequently reported as the most significant [16,21-24], but was excluded in the current study. The remaining findings are elaborated in Table 6.

**Table 6:** Comparison among similar studies. ACS = acute coronary syndrome; CAD = coronary artery disease; CAG = coronary angiography; HbA1c = glycated hemoglobin; HDL = high-density lipoprotein; IHD = ischemic heart disease; LAD = left anterior descending; LDL = low-density lipoprotein; LV = left ventricular; MVD = multivessel disease; NSTEMI = non-ST-segment elevation myocardial infarction; PCI = percutaneous coronary intervention; STEMI = ST-segment elevation myocardial infarction; SVD = single-vessel disease; TG = triglycerides; Trop = troponin I

Serial number	Parameter	Muthu et al. [16]	Bana et al. [21]	Prajapati et al. [22]	Current study
1	Age of inclusion	≤40 years	PCAD: 41–59 years; very PCAD: <40 years	≤40 years	≤45 years
2	Sample Size	69	PCAD: n = 420; very PCAD: n = 79	109	22
Epidemiology:					
1	Male preponderance	87%	Not explicitly mentioned in the text (for PCAD/very PCAD groups)	89.9%	82%
2	Obesity	7.2%	63.5%	Overweight: 36.7%; obese: 5.5%	18%
3	Hypertension	21.7%	41.7%	19.3%	9%
4	Diabetes	24.6%	32.5%	25.7%	22.7%
5	Positive Family history of IHD	11.6%	41.9%	17.4% (PCAD)	27.3%
Clinical presentation and echocardiography findings					
1	STEMI	72.5%	48.7%	82.6%	40.9%
2	NSTEMI (including evolved STEMI and unstable angina)	27.5%	45.1%	17.5%	45.4%
3	LV dysfunction	22%	23.8%	Not explicitly mentioned in the text	50%
Biochemical profiling:					
1	Prediabetes level HbA1c (5.5–6.4%)	49.3%	Not explicitly mentioned in the text	Not explicitly mentioned in the text	50%
2	Diabetic range HbA1c (≥6.5%)	24.6%	Not explicitly mentioned in the text	Not explicitly mentioned in the text	18.2%
3	Hyperhomocysteinemia >15 µmol/L	18.8%	Not explicitly mentioned in the text	Not explicitly mentioned in the text	50%
4	Dyslipidemia	Not explicitly mentioned in the text	Not explicitly mentioned in the text	Not explicitly mentioned in the text	50%
5	Elevated LDL	78.3%	76.9% (LDL >70 mg/dL)	Not explicitly mentioned in the text	50%
6	Low HDL	76.8%	66.3% (HDL <40/50 mg/dL)	Not explicitly mentioned in the text	40.9%
7	Elevated TG	50.7%	46.3% (TG >150 mg/dL)	Not explicitly mentioned in the text	36.4%
Angiographic and PCI profiling:					
1	SVD (including recanalized and non-obstructive CAD)	80%	38.9%	73.4%	72.7%
2	MVD	20.2%	52.1%	22.1%	27.3%
3	LAD involvement	47.8%	Not explicitly mentioned in the text	58.7%	59%
4	Thrombolysis followed by CAG and PCI	44%	Not explicitly mentioned in the text	Not explicitly mentioned in the text	9%
5	Primary PCI	26%	Not explicitly mentioned in the text	Not explicitly mentioned in the text	31.8%

A similar study from South India by Deora et al. [24] reported an incidence of PCAD (age <40 years) of around 10% among patients undergoing coronary angiography. They reported diabetes mellitus and hypertension at 14% and 17%, respectively. While it is known that Southeast Asian males have an earlier onset of CAD [11,25], the INTERHEART study [25] found smoking to be one of the strongest risk factors, followed by diabetes mellitus, hypertension, and a relatively weaker association of BMI. Our findings ran parallel, with diabetes noted in nearly 23% of the patients. However, when prediabetes is taken into consideration, our study had findings similar to Muthu et al. [16], reporting it at nearly 50%. This was supported by the hypothesis that impaired glucose tolerance preceded the onset of PCAD [16].

With respect to clinical presentation, the current study showed STEMI as the most common presentation (41%). This was similar to but lower than that reported by Muthu et al. (72%) [16], Deora et al. (74.5%) [24], and Prajapati et al. (82%) [22]. NSTEMI/unstable angina/delayed presentation of STEMI was reported in 45% in the current study, which is much higher than that reported by either of the aforementioned two studies. Bana et al. reported nearly equal distribution in presentation of STEMI and NSTEMI/unstable angina [21]. Left ventricular dysfunction was noted in 50% of our patients. This is in stark contrast to a majorly normal left ventricle functioning reported by Muthu et al. [16] and Bana et al. [21]. However, Deora et al. [24] reported a mean LVEF of 37% in PCAD patients presenting with STEMI, and 55% in those presenting with NSTEMI.

Biochemical profiling

Hyperhomocysteinemia

The current study found hyperhomocysteinemia in three patients (>50 µmol/L), but 11 patients (50%) had total homocysteine levels >15 µmol/L. This is significantly higher than that described by Muthu et al. (18.8%) [16]. The mean total homocysteine level in the current study (27.2 µmol/L) was comparable to that reported by Prajapati et al. (26.0 µmol/L) [22]. Total homocysteine has been known to contribute to PCAD [26-28] and has been widely acknowledged as an independent marker for CAD [27], with a postulated hypothesis of modulating LpA toxicity via plasmin-modified fibrin binding [29]. Foody et al. proposed a mechanism of LpA/total homocysteine interaction by the inhibition of fibrinolysis and promoting atherothrombosis via ApoA [27], the atherogenic moiety of LpA [30]. Total homocysteine-mediated damage to the vascular matrix and endothelial dysfunction at multiple levels [31], along with pro-oxidative stress, proinflammatory, pro-autoimmune, and procoagulant pathways [32], have also been documented [31]. This has been established in the Indian population as well, with higher levels of total homocysteine being documented in Indians [33], including the Gujarati subgroup [34], and is associated with CAD in young Indians [35].

Dyslipidemia

Similarly, dyslipidemia has also been established as a high-risk factor for CAD as well as PCAD [25]. Dyslipidemia was noted in as many as 50% of the PCAD patients in the current study, which was lower than the nearly 80% dyslipidemia reported in both the STEMI and NSTEMI groups of PCAD patients, reported by Deora et al. [24]. This was lower than the findings of Muthu et al., who reported elevated LDL, elevated triglycerides, and low HDL at 78%, 50%, and 76%, respectively [16]. A registry-based study to evaluate dyslipidemia in PCAD patients concluded dyslipidemia as an important risk factor for PCAD [21]. The mean lipid profile components found in the study are compared against two similar studies in Table 7. It can be seen that the findings of the current study are in accordance with those of Prajapati et al. [22]. The mean LTI in the current study was 20,464, suggestive of a highly atherogenic lipid profile. It is, however, slightly lower than that reported by Prajapati et al. [22], who reported it at 27,809. LDL >130 mg/dL has been included in the risk group, and low HDL has been included in the major ASCVD risk factors mentioned in the Lipid Association of India (LAI) guidelines from 2023 [18]. Similarly, elevated plasma triglycerides are also associated with raised ASCVD risks [18]. Furthermore, after correction for other ASCVD risk factors, elevated non-fasting triglycerides were still associated with myocardial infarctions, IHD, and death in males and females [36]. Dyslipidemia management is the cornerstone of ASCVD prevention, such that more intensive target component levels of medical management for treatment, as well as all levels of prevention, are warranted in the Indian population compared to the Western population [18,37].

**Table 7:** Lipid profile components (comparison of mean values). LpA values were available for 19 of 22 patients. Note: The tabulated data from Bana et al. [21] comprises PCAD (n = 420), very PCAD (n = 79), and non-PCAD (n = 128) groups. ApoB = apolipoprotein B; ApoA1 = apolipoprotein A1; HDL = high-density lipoprotein; LDL = low-density lipoprotein; LTI = Lipid Tetrad Index; TC = total cholesterol; TG = triglycerides; VLDL = very-low-density lipoprotein

Parameter	Bana et al. [21]	Prajapati et al. [22]	Current study
TC (in mg/dL)	159.4	160.3	177.5
LDL (in mg/dL)	106.9	114.7	123.3
VLDL (in mg/dL)	17.7	28	28.3
HDL (in mg/dL)	38	36.5	39.5
TG (in mg/dL)	168.8	139.9	148.3
LTI	Not explicitly mentioned in the text	27,809	20,464
ApoB/ApoA1 ratio	Not explicitly mentioned in the text	0.76	0.60
LpA (in g/L)	Not explicitly mentioned in the text	0.371	0.282

Lipoprotein A and Apolipoprotein A1

While LpA is recognized as pro-atherogenic, ApoA1 is generally considered anti-atherogenic; levels were reported descriptively as part of apolipoprotein profiling. The current study found elevated LpA and ApoA1 in six patients each, of whom two had elevated levels of both. While approximately one-fifth of the global population is estimated to have elevated LpA, the South Asian population accounts for nearly one-third of this global burden [38]. This South Asian population has the highest share of elevated LpA, the highest population-attributable risk, as well as the highest OR for myocardial infarction compared to other ethnicities [38]. LpA, a genetically determined monogenic cardiovascular risk determinant [14,38,39], is hypothesised to be prothrombotic [40], proatherogenic [41] (nearly six times more atherogenic per particle than LDL [38,42]) as well as proinflammatory [43,44], especially in the South Asian population [38]. Around 25% of Indians and other South Asians have elevated LpA levels (≥50 mg/dL) [14], which has been attributed by the LAI as a high-risk parameter for risk stratification [18]. It has even been hypothesised that LpA is likely to have a more significant role in promoting plaque vulnerability and inflammation [38,45,46], rather than the development of a calcific plaque [38,47] along with an increased predisposition toward major adverse cardiac events [38,41,48]. Apolipoprotein is one of the two major protein components of LpA, the other one being ApoB100 [39]. While both these studies were conducted in the Gujarati subpopulation of the SEAP, Prajapati et al. reported a mean LpA of 0.371 g/L [22] (higher than the normal range) compared to the current study (0.282 g/L, i.e., within the normal range). Although the ApoB/ApoA1 ratio has been considered a better index for the likelihood of vascular events, with a ratio >1 suggestive of an increased risk of cardiovascular events [22], the current study, also reported by Prajapati et al. [22], found a mean ratio <1.

Angiographic profiling

The current study found SVD predominance (72%) with LAD being the most commonly involved artery (59%, including the 23% cases where it was a part of MVD). This was similar to other studies that also reported SVD and LAD predominance [16,22,23,49] (Table 6). However, this was in contrast to that reported by Bana et al., who reported MVD predominance (52.1%) in PCAD patients [21]. They reported a finding of SVD in 57% of patients in the ≤40-year age group and 39% of patients in the 41-59-year age group [21]. Similarly, Deora et al. reported SVD predominance (56%) in patients presenting with STEMI, while the NSTEMI/unstable angina group had predominantly insignificant stenosis (43%) [24]. These findings from the current study are in accordance with the general knowledge that younger adults with ACS usually have a less extensive and single-vessel involvement [22,24,50]. However, multiple studies have demonstrated a high proportion of MVD/TVD in young Indians [14], with MVD going up to nearly 80% [51].

Associations

The current study stands out in reporting associations between the clinical, biochemical, and angiographic profiles of PCAD patients without the conventional risk factors. We found that half of the patients with obesity had newly elevated HbA1c and LpA, and a staggering 75% had dyslipidemia. Similarly, more than 80% of the patients with a familial history of IHD also had dyslipidemia. Prajapati et al. reported a significantly higher number of patients with a family history of PCAD than controls [22]. These findings suggest a possible genetic predisposition, not just inherently toward CAD, but probably via a dysregulated metabolism, in turn, generating a thrombogenic environment at a younger age. More than half of STEMI presentations had dyslipidemia, as well as newly diagnosed prediabetic HbA1c levels. Furthermore, more than half of the patients with elevated LpA and elevated ApoA1 presented with acute and delayed STEMI, respectively. Bana et al. reported significantly higher VLDL, triglycerides, and non-HDL cholesterol levels in patients with PCAD and very PCAD compared to non-PCAD patients undergoing PCI [21]. These findings further support the above-mentioned hypothesis, considering an 80-90% genetic determination, variation, and isoform size of the plasma LpA, which makes it nearly immune to alteration by diet and lifestyle [38].

With respect to angiographic associations, we found that more than 75% of obese patients and those with a family history of IHD had SVD. Suri et al. reported similar findings with nearly half of SVD and 75% of TVD patients being obese [49]. However, their finding of <5% of SVD patients having a family history of CAD was in contrast to our results. The current study showed that half of the STEMI and NSTEMI presentations had SVD. This is similar to the findings of Deora et al. [24], who reported 56% of STEMI patients had SVD. While the current study found 50% of NSTEMI presentations to have MVD, Deora et al. reported double and triple vessel involvement in just over 25% of the NSTEMI presentations, with insignificant lesions forming a majority [24]. Additionally, the current study also found elevated LDL levels in half of the patients with SVD as well as MVD. Lastly, 83% of patients with elevated LpA levels had SVD. Although counterintuitive interactions of LpA and diabetes/insulin resistance have been described [14], the current study found three patients with SVD and two patients with MVD to have elevated HbA1c levels.

Biochemical abnormalities reported herein reflect the selected study cohort and should not be interpreted as background population prevalence. LTI was reported descriptively and lacks validated outcome-based thresholds in this setting.

However, among all the above-mentioned studies evaluating PCAD, the current study has a distinctive feature of excluding patients with one of the most prevalent and established risk factors in the form of tobacco consumption, thereby focusing more on the non-modifiable predispositions.

Limitations 

A small sample size, albeit a very select subgroup of an inherently predisposed population, remains a primary limitation. Biochemical parameters were measured during acute coronary presentation and may have been influenced by acute-phase effects. Waist circumference was not assessed. Family history was self-reported. LpA assay standardization and isoform sensitivity were not evaluated. Given the cross-sectional design, temporal relationships cannot be established. Future multicentric longitudinal studies with control groups are required to validate these findings.

## Conclusions

While it has become nearly irrefutable that smoking and/or tobacco consumption is one of the most common risk factors predisposing young adults to CAD, findings from the current study (which excluded such conventional risk factors) show that among the SEAP, a combination of genetic predisposition to biochemical derangements, such as earlier onset of dysregulated metabolic milieu of glycemic and lipidemic alterations along with a strong familial tendency, may be playing a far more under-recognized role than probably acknowledged at the moment. Although the current study has a smaller sample size in a very focal, ethnolinguistic subgroup of the SEAP, these findings hold significance considering the exclusion of conventional risk factors. However, larger multicentric studies from a more diversified population, including genetic analysis and/or Polygenic Risk Scores, may add significant value to understanding the pathophysiology of the significantly higher predisposition in the SEAP, as well as in identifying risk factors and mitigating them at the earliest in suspected individuals.
